# The Joanna Briggs Institute approach for systematic reviews

**DOI:** 10.1590/1518-8345.2885.3074

**Published:** 2018-11-14

**Authors:** Wendel Mombaque dos Santos, Silvia Regina Secoli, Vilanice Alves de Araújo Püschel

**Affiliations:** 1 Empresa Brasileira de Serviços Hospitalares, Hospital Universitário de Santa Maria, Santa Maria, Rio Grande do Sul, Brazil.; 2 Universidade de São Paulo, Escola de Enfermagem, São Paulo, SP, Brazil.

In the last decades, systematic and integrative review studies have occupied important space in high impact journals. Reviews, in theory, offer the best evidence on certain topics; are original studies; and do not require approval in Research Ethics Committees (REC). The need to support practices, especially clinical and educational, in contrast to the dispensation of the REC, and the limited knowledge of International Centers Specialized in Revisions guidelines have favored the dissemination of questionable quality works.

In 2017, a published article reported-on the basis of an integrative review using the Joanna Briggs Institute (JBI) method-the gains perceived by health students and professionals in the use of clinical simulation using dramatization^1^. These scholars used the term “integrative review”; however, a close examination of their work reveals that they began it as a “comprehensive review” (using both quantitative and qualitative questions) and completed it as a rudimentary “scoping review.”

The term “integrative review” has been used loosely, and certain authors have considered reviews of any kind (including those of variant study designs; such as, experimental, observational, and descriptive) to be integrative^2^. However, other authors suggest that integrative review requires a synthesis of theoretical studies, i.e., something more than mere empirical evidence^2^. JBI provides formal guidance for ten types of reviews; however, none of them refer to how an integrative review should be performed^3^.

The systematic reviews of the JBI are based on the model of evidence-based healthcare, which does not concern exclusively with effectiveness, rather focuses on basing practice on the best available evidence, and is adaptable to the diverse origins of problems in health care, using a diverse range of research methodologies to generate evidence appropriate to the issue^3^. JBI considers that health professionals require evidence to substantiate a wide range of activities and interventions, and while making clinical decisions, they must examine whether their approach is feasible, appropriate, meaningful, and effective^3^ ^-^ ^4^.

JBI systematic reviews are aimed at providing a comprehensive and unbiased synthesis of large numbers of relevant studies within the confines of a single document by using rigorous and transparent methods^4^. Such a systematic review seeks to synthesize and summarize existing knowledge rather than to create new knowledge^5^. This produces decision-making that considers the feasibility, appropriateness, meaningfulness, and effectiveness of healthcare practice^4^ ^-^ ^5^. The best available evidence, the context in which care is delivered, the individual patient, and the expertise and professional judgment of the health professionals play a role in this process^4^ ^-^ ^6^.

Thus, we recommend using JBI methodology to conduct systematic reviews of the following items: effectiveness, experiential (qualitative), cost/economic evaluation, prevalence and/or incidence, diagnostic text accuracy, etiology and/or risk, expert opinion/policy, psychometric, prognostic, and methodology^6^.

The credibility of the knowledge produced and the usefulness of the product generated, based on the review studies, according to the epidemiological delineations, is closely related to methodological rigor, an aspect that can be qualified through the guidelines of the Review Centers.
